# Metabolic Score for Insulin Resistance Is Inversely Related to Incident Advanced Liver Fibrosis in Patients with Non-Alcoholic Fatty Liver Disease

**DOI:** 10.3390/nu14153039

**Published:** 2022-07-24

**Authors:** Jun-Hyuk Lee, Yu-Jin Kwon, Kyongmin Park, Hye Sun Lee, Hoon-Ki Park, Jee Hye Han, Sang Bong Ahn

**Affiliations:** 1 Department of Family Medicine, Nowon Eulji Medical Center, Eulji University School of Medicine, Seoul 01830, Korea; swpapa@eulji.ac.kr; 2 Department of Medicine, Hanyang University Graduate School of Medicine, Seoul 04763, Korea; minitel21c@naver.com (K.P.); hoonkp@hanyang.ac.kr (H.-K.P.); 3 Department of Family Medicine, Yongin Severance Hospital, Yonsei University College of Medicine, Yongin 16995, Korea; digda3@yuhs.ac; 4 Department of Family Medicine, Hanyang University College of Medicine, Seoul 04763, Korea; 5 Biostatistics Collaboration Unit, Department of Research Affairs, Yonsei University College of Medicine, Seoul 06273, Korea; hslee1@yuhs.ac; 6 Department of Internal Medicine, Nowon Eulji Medical Center, Eulji University School of Medicine, Seoul 01830, Korea

**Keywords:** liver fibrosis, insulin resistance, incidence, Korean, non-alcoholic fatty liver disease

## Abstract

We determined the relationships between metabolic score for IR (METS-IR), triglyceride-glucose (TyG) index, and homeostatic model assessment for IR (HOMA-IR) and incident advanced liver fibrosis (ALF) and assessed the abilities of the three IR indicators to predict ALF in patients with non-alcoholic fatty liver disease (NAFLD) in adults with NAFLD who were aged 40–69 years old. Among 2218 participants with NAFLD at baseline, the areas under the receiver operating characteristic curve for predicting ALF of the METS-IR was 0.744 (0.679–0.810), significantly higher than that of TyG index (0.644 (0.569–0.720)) or that of HOMA-IR (0.633 (0.556–0.710)). Among 1368 patients with NAFLD and without ALF at baseline, 260 (19.0%) patients with NAFLD progressed to ALF during the 16-year follow-up period. Multivariable Cox proportional hazard regression analysis revealed that the adjusted hazard ratio (95% confidence interval) for incident ALF in the highest tertiles of METS-IR, TyG index, and HOMA-IR compared with the lowest tertile of each IR indicator were 0.5 (0.36–0.91), 0.7 (0.49–1.00), and 1.01 (0.71–1.42), respectively. METS-IR was inversely associated with ALF in patients with NAFLD, which cautiously suggests that the risk of ALF may need to be evaluated when metabolic parameters improve in individuals with NAFLD.

## 1. Introduction

Non-alcoholic fatty liver disease (NAFLD) is the most common cause of chronic liver diseases [1]. NAFLD comprises a broad clinical spectrum of progression, including non-alcoholic fatty liver without necrotic inflammatory entity and non-alcoholic steatohepatitis, which could be accompanied by hepatic injury inflammation and fibrosis [2].

Liver fibrosis is characterized by abnormal accumulation of extracellular matrix and hyperplasia of connective tissue. Liver fibrosis results from chronic liver damage and could further develop into liver cirrhosis and hepatocellular carcinoma [3]. Chronic liver diseases accompanying liver fibrosis are also associated with increasing morbidity and mortality worldwide [4]. Although early-stage liver fibrosis is known to be reversible because of the balance of pro-fibrosis and anti-fibrosis mechanisms [3], early detection of liver fibrosis is difficult because of its non-symptomatic nature, and effective treatments for liver fibrosis remain unclear. The aims for treatment of liver fibrosis are linked to the main pathogenesis for reducing hepatic fibrosis, inhibiting inflammation, inhibiting oxidative stress, inhibiting hepatocyte apoptosis, inhibiting hepatic stellate cells activation and proliferation, and inhibiting extracellular matrix production [5]. The imbalance between production of tumor necrosis factor-alpha, interleukin-6, leptin, free fatty acids and adiponectin cause insulin resistance and inflammation that account for major pathophysiology of liver fibrosis in fatty liver [6]. Therefore, current therapeutic treatment for liver fibrosis focuses on reducing insulin resistance and improving insulin sensitivity [6].

To date, insulin resistance (IR) is a pathophysiological hallmark of NAFLD and many other metabolic diseases [7]. Although the hyperinsulinemic–euglycemic clamp technique is the gold standard for evaluating insulin sensitivity in humans [8], this tool is not applicable in large epidemiological studies because of its invasive and impractical nature. Therefore, homeostatic model assessment for IR (HOMA-IR) and other non-insulin-based indices for assessing IR, such as the triglyceride-glucose (TyG) index and metabolic score for IR (METS-IR), were developed for use instead of the hyperinsulinemic–euglycemic clamp technique. Several studies have reported the usefulness of three indicators separately as surrogates for NAFLD [9,10,11]. However, the relationship between markers related to IR and liver fibrosis is more complex. Although there is evidence that insulin resistance is associated with liver fibrosis [12], the effect of insulin resistance on lipid metabolism is opposite to that of liver fibrosis on lipid metabolism. During progression of liver fibrosis, decreased total cholesterol (TC), low-density lipoprotein cholesterol (LDL-C), triglycerides (TGs), and high-density lipoprotein cholesterol (HDL-C), due to decreased synthesis by the liver, have been noted [13]. This implies that the lipid profile may be used as an indicator to reflect the progression of liver fibrosis. On the other hand, insulin resistance leads to decreased TG clearance via increased apolipoprotein C level, which modulates plasma TG level through lipoprotein lipase-independent mechanisms [14]. Sinceboth the TyG index and METS-IR include the blood lipid profiles in the formula, while HOMA-IR does not include the blood lipid profile in the formula, the TyG index and METS-IR could be more likely to reflect not only insulin resistance but also liver fibrosis than HOMA-IR.

Based on these perspectives, we hypothesized that IR markers, especially METS-IR, which contains two lipid markers, would be inversely related to the incidence of advanced liver fibrosis (ALF) among patients with NAFLD, usually with insulin resistance, based on the evidence from other studies [15,16]. We also aimed to evaluate the relationships between the METS-IR, TyG index, and HOMA-IR and incident ALF and further assess the abilities of the three IR indicators to predict progression to ALF in patients with pre-existing NAFLD.

## 2. Materials and Methods

### 2.1. Study Population

We used the data from Korean Genome and Epidemiology Study (KoGES) conducted by the Korea Centers for Disease Control and Prevention [17]. The KoGES-Ansan and Ansung is a longitudinal prospective cohort study that consited adults aged 40 to 69 years. The KoGES-Ansan and Ansung cohort began in 2001–2002 (baseline study) and ended in 2017–2018 (8th follow-up).

Supplementary Figure S1 shows the study population selection process. From a total of 10,030 participants who participated in the baseline survey, we excluded (1) those with a history of hepatitis (*n* = 423); (2) men whose alcohol consumption was ≥30 g/day or women whose alcohol consumption was ≥20 g/day (*n* = 964); (3) insufficient data to calculate the NAFLD-liver fat score (*n* = 276); (4) insufficient data to calculate the METS-IR, TyG index, and HOMA-IR (*n* = 7); and (5) participants without NAFLD at baseline (*n* = 6142). From the remaining 2218 participants with NAFLD at baseline, we compared the abilities to predict incident ALF of the METS-IR, TyG index, and HOMA-IR. In addition, to compare the abilities of the three different insulin resistance indices studied to predict the incidence of ALF, we analyzed the data from 1368 NAFLD participants without ALF at baseline who were followed-up at least once after the baseline survey after applying the following exclusion criteria: (1) participants with NAFLD and ALF at baseline (*n* = 70) and (2) participants who were not followed-up after the baseline survey (*n* = 780).

Informed consents were obtained from all participants. The protocol of this study was approved by the institutional review of board of Nowon Eulji Medical Center (IRB number: 2022-01-016).

### 2.2. Measurements

The parameters necessary for evaluating IR, NAFLD, and ALF included fasting plasma glucose (FPG), serum TG, serum HDL-C, serum insulin, body mass index (BMI), waist circumference, systolic blood pressure (SBP), diastolic blood pressure (DBP), hypertension, diabetes mellitus (DM), dyslipidemia, serum aspartate aminotransferase (AST), serum alanine aminotransferase (ALT), and whole blood platelet count. Laboratory variables related to IR, NAFLD, and ALF included serum TC, gamma-glutamyl transpeptidase (GTP), total bilirubin, albumin, and C-reactive protein (CRP). In addition, lifestyle factors related to IR, NAFLD, and ALF included smoking status, alcohol-drinking status, physical activity level, and diet.

BMI is a value is calculated by dividing the body weight by height squared (kg/m^2^). Waist circumference (cm) was measured in the horizontal plane midway between the lowest rib and iliac crest. Abdominal obesity was defined as ≥90 cm (men) or ≥85 cm (women) according to the cut-off point for the definition of abdominal obesity in the Korean population [18]. SBP and DBP were obtained after 5 min of rest in the sitting position. We calculated mean blood pressure (MBP) as DBP + 1/3 × (SBP − DBP).

Blood samples were collected after ≥8 h of fasting and analyzed using a Hitachi 700–110 Chemistry Analyzer (Hitachi Co., Tokyo, Japan). Whole blood platelet count, FPG levels, serum insulin, TC, HDL-C, TG, AST, ALT, GTP, total bilirubin, albumin, and CRP levels were measured. LDL-C was calculated according to the Friedewald equation in the case of TG < 400 mg/dL.

DM was determined as an FPG level ≥ 126 mg/dL, post 2-hour plasma glucose level after the 75 g oral glucose tolerance test of ≥200 mg/dL glycosylated hemoglobin ≥ 6.5%, treatment with anti-diabetic medications, or treatment with insulin therapy. Hypertension was determined as SBP ≥ 140 mmHg, DBP ≥ 90 mmHg, or treatment with anti-hypertensive medications. Dyslipidemia was determined as serum TC ≥ 240 mg/dL, LDL-C ≥ 160 mg/dL, HDL-C < 40 mg/dL, TG ≥ 200 mg/dL, or treatment with lipid-lowering agents. Metabolic syndrome was defined as the presence of three or more of the following factors: (1) abdominal obesity; (2) FPG ≥ 100 mg/dL or treatment with anti-diabetic medications or insulin therapy; (3) SBP ≥ 130 mmHg, DBP ≥ 85 mmHg, or treatment with anti-hypertensive medications; (4) serum TG ≥ 150 mg/dL or treatment with lipid-lowering medications; and (5) serum HDL-C < 40 mg/dL (men) or <50 mg/dL (women).

Smoking status was categorized into four groups: never smoker, former smoker, some day smoker, or every day smoker. A current drinker was defined as any participant who drank < 30 g of alcohol per day (men) or <20 g of alcohol per day (women). Physical activity using metabolic equivalent of task (MET)-hours per week (METs-h/week), which was obtained from the participant’s report on hours spent on sleep and five types of physical activities according to intensity, including sedentary, very light, light, moderate, and heavy, and corresponded to 0, 1.5, 3, 5, and 7 METs, respectively. The physical activity was classified into three groups; low (<7.5 MET-h/week), moderate (7.5–30 MET-h/week), and high (>30 MET-h/week). A validated 103-item food frequency questionnaire was used for dietary surveillance. Total energy intake (kcal/day), carbohydrate intake (g/day), protein intake (g/day), fat intake (g/day), and vitamin E intake (mg/day) were calculated.

### 2.3. Assessment of Insulin Resistance

The METS-IR, TyG index, and HOMA-IR were calculated as follows [19,20,21]:

(1) METS-IR = ln [2 × FPG (mg/dL) + fasting serum TG (mg/dL)] × BMI (kg/m^2^)/ln [HDL-C (mg/dL)].
(2) TyG index = ln [fasting serum TG (mg/dL) × FPG (mg/dL)/2].
(3) HOMA-IR = [fasting serum insulin (μU/mL) × FPG (mg/dL)/405].

### 2.4. Assessment of NAFLD

NAFLD was determined using the NAFLD-liver fat score. The NAFLD-liver fat score was defined by the following formula [22]:

NAFLD-liver fat score = −2.89 + 1.18 × metabolic syndrome (Yes: 1, No: 0) + 0.45 × DM (Yes: 2, No: 0) + 0.15 × insulin in µIU mL + 0.04 × AST in U/L − 0.94 × AST/ALT.

A NAFLD-liver fat score > −0.640 was considered as NALFD [22].

### 2.5. Assessment of ALF

We used a surrogate marker for assessing liver fibrosis: the fibrosis-4 (FIB-4) index, a metabolic indicator for reliably predicting liver fibrosis [23]. The formula for FIB-4 index is as follows [24]:

FIB-4 index = (age [years] × AST [U/L])/(platelet [10^9^/L] × $\sqrt{\mathrm{ALT}\;\left[U/L\right]}$)

To apply a cutoff point for the definition of ALF in patients with NAFLD, we set the FIB-4 index to ≥2.67 as having ALF, on the basis of a previous study [25].

### 2.6. Statistical Analysis

All of the data are presented as number (percentage, %) for categorical variables or the mean ± standard deviations or median (25th, 75th) for continuous variables. We used independent *t*-test for continuous variables and the chi-square test for categorical variables to compare the clinical characteristics of NAFLD participants with or without ALF at baseline as well as the baseline characteristics of NAFLD participants without ALF at baseline.

Among the NAFLD participants with or without liver fibrosis at baseline, the area under the receiver operating characteristic (ROC) curves (AUC) was used to compare the abilities of three different IR indices to predict the prevalence of ALF. The Youden index was used to calculate the cutoff point for predicting the ALF of each IR index [26].

Cox proportional hazard spline curves using data from the NAFLD participants without ALF at baseline were prepared to determine the dose–response relationship between each IR index and incident ALF. The hazard ratio (HR) with 95% confidence interval (CI) for incident ALF of each IR index per increment was calculated using the Cox proportional hazards regression analysis. After dividing METS-IR, TyG index, and HOMA-IR into tertiles (T) for each index, we calculated the HR with 95% CI for incident ALF of the highest tertile (T3) and middle tertile (T2) compared to the lowest tertile (T1) of each IR index using the Cox proportional hazards regression analysis. We adjusted for sex, age, BMI, physical activity, smoking status, drinking status, total energy intake, and vitamin E intake in model 1. We further adjusted for MBP, serum TC level, and serum CRP level in model 2 and additionally adjusted for serum ALT level in model 3.

All statistical analyses were performed in SAS statistical software (version 9.4; SAS Institute Inc., Cary, NC, USA) and R (version 4.1.1; R Foundation for Statistical Computing, Vienna, Austria). *p* < 0.05 was regarded significant.

## 3. Results

### 3.1. Characteristics of the Study Population

The characteristics of 2218 participants with or without ALF at baseline are shown in Supplementary Table S1. The proportion of men was higher in the participants with ALF than without (67.1% vs. 42.4%, *p* < 0.001). The mean age of the participants was higher in those with liver fibrosis than without (60.1 y vs. 54.1 y, *p* < 0.001). The mean values of BMI, waist circumference, whole blood platelet count, serum TC, LDL-C, albumin levels, METS-IR, TyG index, and HOMA-IR; the median values of serum insulin and TG levels; and the proportion of participants with dyslipidemia were significantly lower in the participants with ALF than in those without. The mean values of serum HDL-C level; the median values of serum AST, ALT, gamma-GTP, and total bilirubin levels; the FIB-4 score; and the proportions of current drinkers and everyday smokers were significantly higher in the participants with ALF than in those without.

Table 1 presented the characteristics of the NAFLD patients according to incident ALF status. The mean age, waist circumference, MBP, serum AST, ALT, and FIB-4 score; the median values of serum gamma-GTP; and the proportion of the participants with high physical activity were significantly higher in the participants with newly developed ALF. The mean values of whole blood platelet count, serum TC, LDL-C, albumin levels, and METS-IR; the TyG index; and the proportion of everyday smokers were significantly lower in the participants with newly developed ALF than in those.

### 3.2. Comparison of Predictive Power for Incident ALF of METS-IR, TyG Index, and HOMA-IR at Baseline

**Figure 1:**
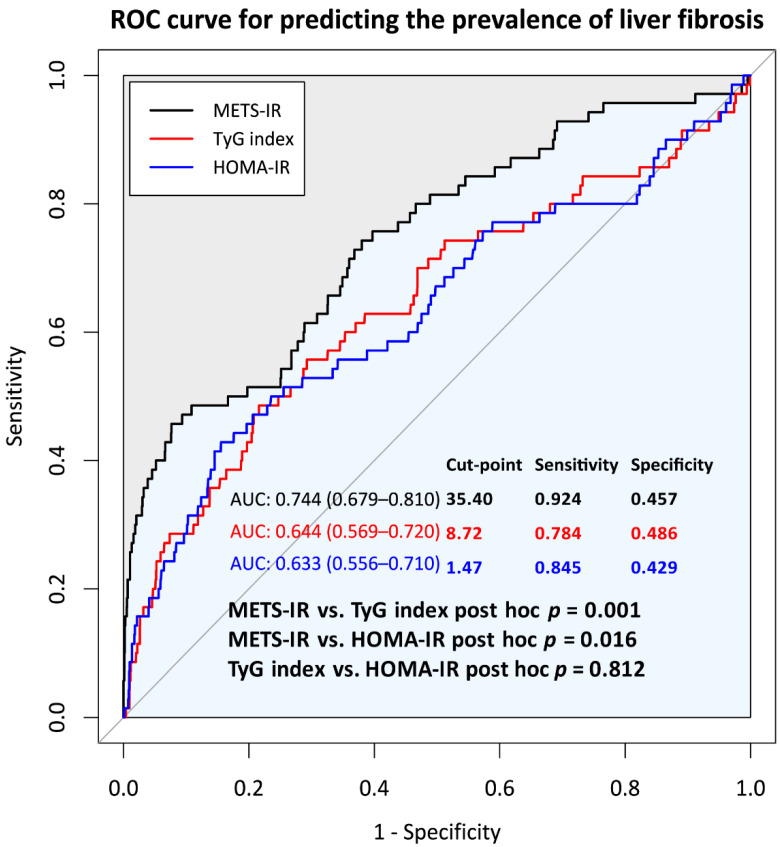
Comparison of the abilities to predict ALF of three different insulin resistance indices. The gray line shows the chance level performance. Abbreviation: ALF, advanced liver fibrosis.

Figure 1 shows a comparison of ROC curves for predicting the incidence rate of ALF by the METS-IR, TyG index, HOMA-IR. The AUCs (range) of the METS-IR, TyG index, and HOMA-IR for predicting ALF were 0.744 (0.679–0.810), 0.644 (0.569–0.720), and 0.633 (0.556–0.710), respectively, with significant differences between the METS-IR and TyG index as well as between METS-IR and HOMA-IR. No significant difference in the predictive powers between the TyG index and HOMA-IR was noted. The cutoff points for incident ALF of the METS-IR, TyG index, and HOMA-IR were 35.4, 8.72, and 1.47, respectively.

### 3.3. Longitudinal Relationships between the METS-IR, TyG Index, and HOMA-IR and Incident ALF in Patients with NAFLD

The mean follow-up time was 15.8 years. The incidence rate for ALF per 2 years ranged from 1.24 to 3.14 (Table 2).

**Figure 2:**
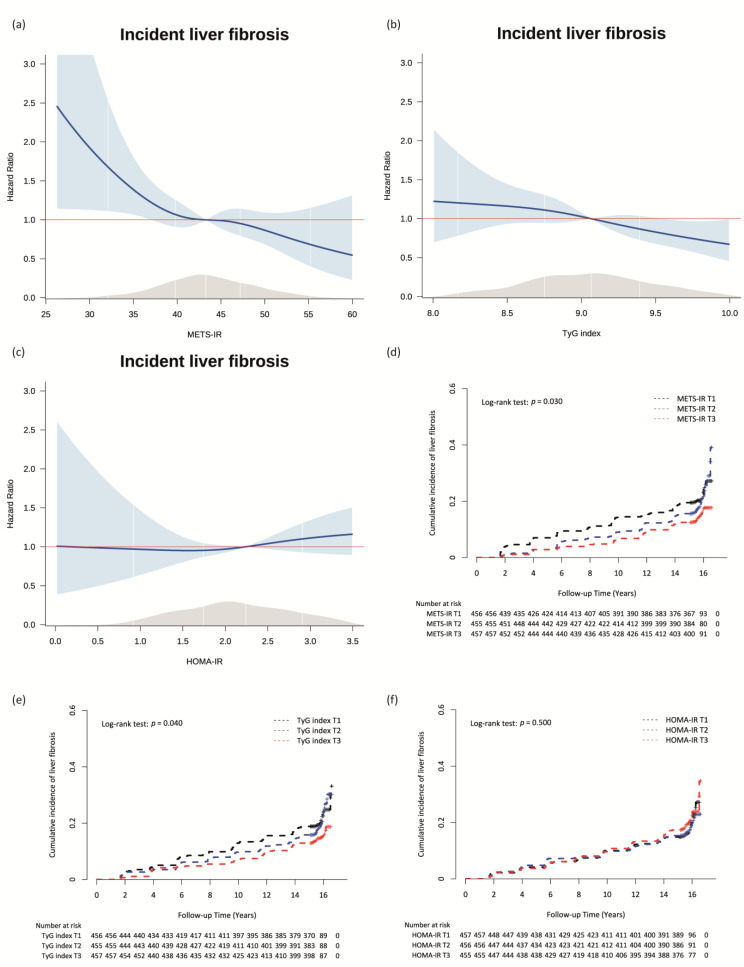
Relationships between the METS-IR, TyG index, and HOMA-IR and incident ALF. Cox proportional hazards spline curve showing the dose-response relationships between incident ALF and (**a**) METS-IR, (**b**) the TyG index, and (**c**) HOMA-IR. The red line indicates a hazard ratio of 1. The blue lines represent the hazard ratio for incident ALF of METS-IR, TyG index, and HOMA-IR. The blue area represents 95% confidence interval for hazard ratio. The grey area represents density of values. Kaplan-Meier curves showing the cumulative incidence rate of ALF according to the tertiles of (**d**) the METS-IR, (**e**) TyG index, and (**f**) HOMA-IR. Abbreviations: METS-IR, metabolic score for insulin resistance; TyG, triglyceride-glucose; HOMA-IR, homeostatic model assessment for insulin resistance; ALF, advanced liver fibrosis.

The Cox proportional hazard spline curves showing the dose–response relationships between the METS-IR, TyG index, and HOMA-IR and incident ALF are shown in Figure 2a–c. Inverse dose–response relationships were observed between the METS-IR and TyG index and incident ALF in patients with NAFLD (Figure 2a,b). The dose-response relationship between HOMA-IR and incident ALF was a nearly flat U-shape (Figure 2c).

Figure 2d–f shows the cumulative incidence rate of incident ALF according to the tertiles of each IR index using Kaplan–Meier curves. The T3 of the METS-IR and TyG index were associated with significantly lower incidences of ALF than the referent T1s of two IR indices during the follow-up period (Figure 2d,e). There were no significant associations among the tertiles of HOMA-IR with the incident rate of ALF (Figure 2f).

Table 3 presents the Cox proportional hazard regression model for incident ALF according to the tertiles of each IR index. During a total of 19,939.4 person-years of follow-up, there were 260 (19.0%) cases of newly developed ALF among the participants with NAFLD. The incidence rate per 1000 person-years was 13.0. METS-IR was significantly associated with the incidence of ALF (HR = 0.67, 95% CI = 0.49–0.90). This significant association remained in all adjusted models. In model 3, the adjusted HR (95% CI) for incident ALF of the T3 of METS-IR compared with the referent lowest T1 of METS-IR was 0.59 (0.37–0.94). The T3 of the TyG index was also significantly and inversely associated with incident ALF compared with the T1 of the TyG index in model 1 (HR = 0.66, 95% CI = 0.47–0.92); however, the significant association was attenuated in models 2 and 3 (model 2: HR = 0.73, 95% CI = 0.52–1.03; model 3: HR = 0.74, 95% CI = 0.53–1.04). HOMA-IR was not associated with incident ALF.

Supplementary Table S2 shows the Cox proportional hazard regression analyses for incident ALF of three different IR indices. The HRs with 95% CIs of METS-IR, TyG index, and HOMA-IR for incident ALF per increment were 0.97 (0.95–0.99), 0.71 (0.56–0.91), and 1.02 (0.98–1.06) in the unadjusted model, respectively. The adjusted HRs with 95% CIs for incident ALF of the METS-IR, TyG index, and HOMA-IR per increment were 0.92 (0.88–0.96), 0.66 (0.49–0.89), and 1.03 (0.95–1.11) in model 3, respectively.

**Figure 3:**
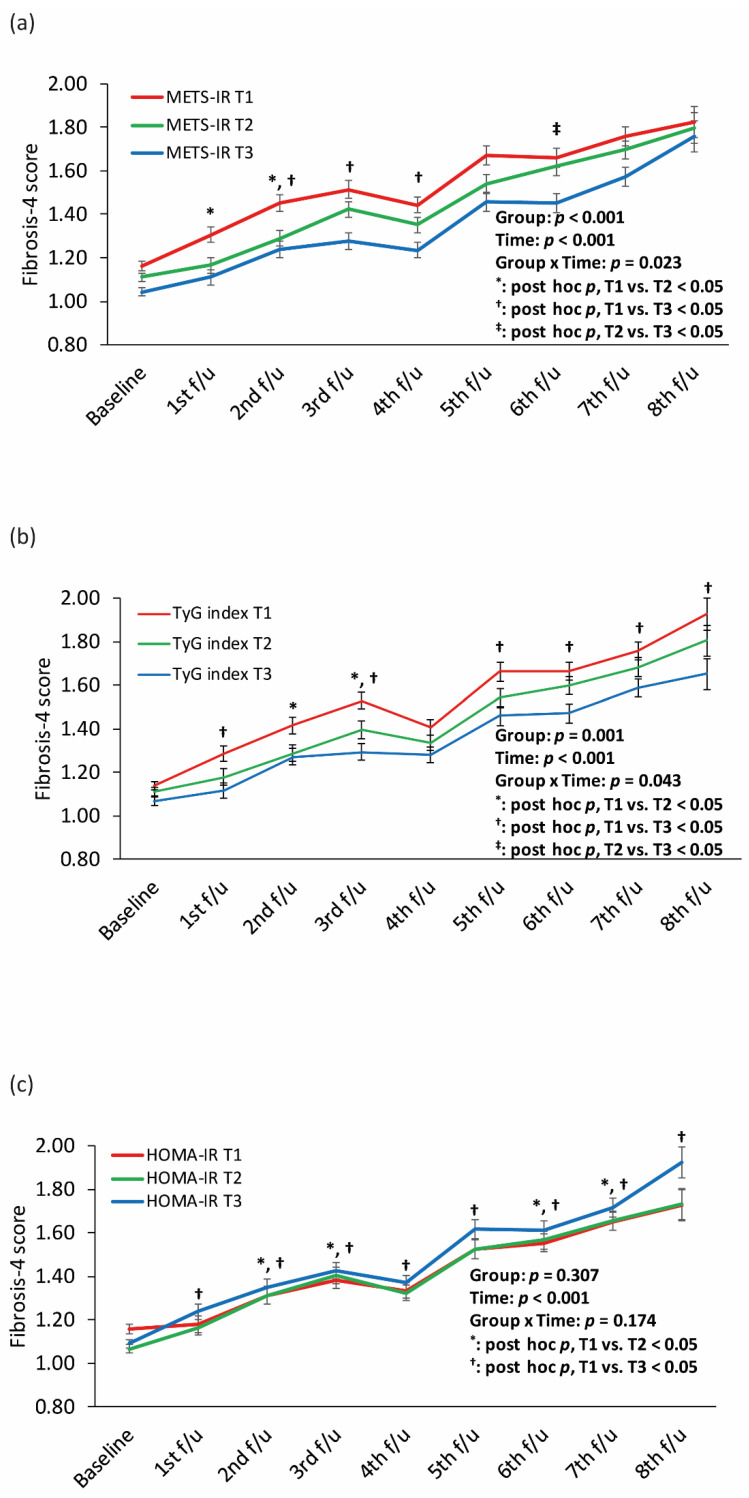
Longitudinal changes in the FIB-4 score according to the tertiles of (**a**) METS-IR, (**b**) the TyG index, and (**c**) HOMA-IR. Abbreviation: FIB-4, fibrosis-4; METS-IR, metabolic score for insulin resistance; TyG, triglyceride-glucose; HOMA-IR, homeostatic model assessment for insulin resistance.

Figure 3 shows longitudinal changes in the FIB-4 score according to the tertiles of each IR index. The T1 of the METS-IR and TyG index had the highest FIB-4 score during all follow-up periods, followed by the T2 and T3, respectively, with significant interactions between group and time. The T3 of HOMA-IR showed the highest FIB-4 score during the follow-up period except for the baseline, without a significant interaction between group and time.

Supplementary Table S3 shows Cox proportional hazard regression analyses for incident ALF according to the tertiles of each IR index in participants with and without diabetes. In participants without DM, there were significant association between METS-IR, TyG index, and incident ALF. However, in participants with DM, there was no significant association between METS-IR, TyG index, and incident ALF.

## 4. Discussion

We found that METS-IR was inversely associated with incident ALF in patients with NAFLD. Furthermore, the predictive value for ALF was highest for METS-IR than for the TyG index and HOMA-IR at the baseline survey. Previous studies have shown that the presence of hyperglycemia was a strong independent predictor of liver fibrosis [27] and have suggested the HOMA-IR may facilitate ALF [28]. However, our results did not show a significant association between HOMA-IR and incident ALF. Although T3 of HOMA-IR showed the highest FIB-4 score during the longitudinal follow-up period, there was no significant interaction between group and time. Chronic liver injury might affect glucose metabolism, which is responsible for glucose synthesis and storage [29]. Patients with DM included in this study also could affect the result. In the subgroup analysis according to the presence of diabetes (Supplementary Table S3), there was no significant association between METS-IR, TyG, and HOMA-IR and incident ALF in participants with diabetes. Although the exact reasons for such discrepancy were unclear, diabetes was characterized by the dysregulation of glucose homeostasis. Therefore, these associations might be attenuated in the participants with diabetes. Further studies are warranted to find more useful markers to predict ALF in participants with diabetes.

There are several possible mechanisms that could support our finding that METS-IR was inversely associated with ALF in patients with NAFLD.

Although the most common finding in NAFLD is high serum TG level, and abnormalities of lipid metabolism have been reported in patients with NAFLD in terms of increased levels of serum TC and/or serum TG [30], serum TG levels decreased as liver disease progressed to liver fibrosis [31]. As triglycerides, which are normally the principal source of lipids in the liver [31], the fat mass in the liver may decrease when the liver fibrosis progresses [32]. Therefore, we additionally used a linear mixed model to compare changes in the serum TG level according to the tertiles of METS-IR, which showed that the mean serum TG level of METS-IR T3 was highest during the follow-up period, followed by T2 and T1 (Supplementary Figure S2).

Due to the strong relationship between over-nutrition, obesity, and the development of NAFLD, reducing energy intake and weight loss are currently considered to be the fundamental strategy for treatment of NAFLD and its complications. However, poor nutritional status in patients with hepatic disorders has been under-recognized. Patients with hepatic diseases are vulnerable to developing malnutrition because of hypermetabolism, malabsorption, and altered nutrient metabolism in the liver [33]. In the current study, we found that the BMI was lower in patients with ALF than in those without ALF at the baseline survey. We also found a significant inverse association between METS-IR and ALF. Furthermore, the predictive power of METS-IR was significantly higher than those of TyG index and HOMA-IR. The inclusion of BMI reflecting nutritional status might explain why METS-IR better reflects the ALF prevalence in patients with NAFLD. To test this hypothesis, we performed a linear mixed-model analysis to compare the longitudinal change in BMI of participants with and without ALF at baseline, which revealed that the group with ALF had a consistently lower average BMI than the group without ALF during the follow-up period (Supplementary Figure S3). In addition, in the linear mixed-model analysis comparing tertiles of METS-IR among those without ALF at baseline, the T3 of METS-IR showed the highest BMI during the follow-up period followed by T2 and T1 (Supplementary Figure S4).

Our study had several limitations. First, histological diagnosis of fatty liver and liver fibrosis was not conducted. Second, metabolic parameters assessed for IR were not updated during the follow-up period, and thus, baseline exposures might not reflect over time. Third, we could not obtain detailed information about individuals’ drug-use history and symptoms related to drug-induced liver injury. However, to avoid the possibility of including persons susceptible for drug liver injury, we excluded the participants with a history of hepatitis. Finally, our results could not be generalized to other countries and ethnic groups because the analyzed data were only from middle-aged and older Korean adults. Despite these limitations, a study strength is that this was a large population-based cohort study with a 16-year follow-up period. Moreover, this is the first study to verify the inverse association between METR-IR and ALF as well as compare the abilities of METS-IR, the TyG index, and HOMA-IR to predict incident ALF.

## 5. Conclusions

The current study indicates that METS-IR, unlike the TyG index and HOMA-IR, was inversely associated with incident ALF in patients with NAFLD. This finding may be explained by the impaired lipid metabolism and malnutrition that accompanies progression to liver fibrosis. Therefore, we cautiously suggest that the risk of ALF may need to be evaluated when metabolic parameters improve in individuals with NAFLD. More studies are needed to clarify the underlying mechanism between metabolic parameters and the development of ALF.

## Figures and Tables

**Table 1:** Clinical characteristics of patients with NAFLD according to incident ALF.

Variables.	Did Not Develop ALF	Newly Developed ALF	Total	p *
Number of participants, *n*	1108	260	1368	
Male sex, *n* (%)	460 (41.5%)	126 (48.5%)	586 (42.8%)	0.049
Age, years	52.3 ± 8.0	59.3 ± 7.7	53.6 ± 8.4	<0.001
Waist circumference, cm	88.9 ± 7.3	90.2 ± 7.8	89.1 ± 7.4	0.012
Body mass index, kg/m^2^	26.7 ± 2.8	26.5 ± 3.2	26.7 ± 2.9	0.192
MBP, mmHg	102.5 ± 12.3	104.5 ± 11.5	102.9 ± 12.2	0.017
Current drinker, *n* (%)	430 (39.2%)	102 (40.0%)	532 (39.3%)	0.869
Smoking status, *n* (%)				0.003
Never smoker	719 (65.8%)	146 (57.3%)	865 (64.2%)	
Former smoker	155 (14.2%)	55 (21.6%)	210 (15.6%)	
Some days smoker	19 (1.7%)	10 (3.9%)	29 (2.2%)	
Everyday smoker	200 (18.3%)	44 (17.3%)	244 (18.1%)	
Physical activity		0.031		
<7.5 METs-h/week	719 (65.8%)	146 (57.3%)	865 (64.2%)	
7.5–30 METs-h/week	155 (14.2%)	55 (21.6%)	210 (15.6%)	
≥30 METs-h/week	19 (1.7%)	10 (3.9%)	29 (2.2%)	
Platelets,/mm^3^	287.3 ± 60.5	232.1 ± 52.2	276.8 ± 62.8	<0.001
Glucose, mg/dL	94.9 ± 29.1	93.9 ± 28.0	94.8 ± 28.9	0.617
Insulin, µIU/mL	10.2 (7.8; 12.5)	10.4 (8.2; 13.3)	10.2 (7.8; 12.6)	0.147
Total cholesterol, mg/dL	201.3 ± 34.0	192.4 ± 36.0	199.6 ± 34.6	<0.001
Triglyceride, mg/dL	192.0 (146.5; 260.5)	178.0 (138.0; 225.5)	188.0 (145.0; 253.5)	0.005
HDL cholesterol, mg/dL	39.7 ± 7.9	40.8 ± 9.2	39.9 ± 8.2	0.071
LDL cholesterol, mg/dL	120.3 ± 31.2	113.8 ± 32.1	119.1 ± 31.5	0.004
AST, U/L	31.1 ± 14.5	37.8 ± 17.6	32.4 ± 15.4	<0.001
ALT, U/L	37.2 ± 27.1	40.6 ± 23.8	37.8 ± 26.6	0.047
Gamma-GTP, U/L	26.0 (16.0; 44.0)	30.5 (18.0; 62.0)	27.0 (16.0; 47.5)	0.001
Total bilirubin, mg/dL	0.5 (0.4; 0.7)	0.5 (0.4; 0.7)	0.5 (0.4; 0.7)	0.195
Albumin, g/L	4.3 ± 0.3	4.2 ± 0.3	4.2 ± 0.3	<0.001
CRP, mg/dL	0.18 (0.10; 0.29)	0.19 (0.10; 0.31)	0.18 (0.10; 0.29)	0.429
Total energy intake, kcal/day	1997.1 ± 713.3	1949.0 ± 737.0	1988.0 ± 717.8	0.340
CHO intake, g/day	358.3 ± 129.5	354.2 ± 133.8	357.5 ± 130.3	0.653
Protein intake, g/day	66.3 ± 27.0	63.2 ± 27.4	65.7 ± 27.1	0.109
Fat intake, g/day	31.1 ± 19.0	28.8 ± 19.3	30.7 ± 19.1	0.096
Vitamin E intake, mg/day	9.7 ± 5.8	9.1 ± 5.0	6 ± 5.7	0.112
Diabetes mellitus, *n* (%)	310 (28.0%)	79 (30.4%)	389 (28.4%)	0.485
Hypertension, *n* (%)	662 (59.7%)	178 (68.5%)	840 (61.4%)	0.012
Dyslipidemia, *n* (%)	825 (74.5%)	181 (69.6%)	1006 (73.5%)	0.130
Fibrosis-4 score	1.00 ± 0.34	1.60 ± 0.49	1.11 ± 0.44	<0.001
METS-IR	43.76 ± 5.78	42.65 ± 5.82	43.55 ± 5.80	0.005
TyG index	9.11 ± 0.53	9.01 ± 0.48	9.09 ± 0.52	0.006
HOMA-IR	2.62 ± 2.26	2.78 ± 2.21	2.65 ± 2.25	0.284

* *p*-value was calculated to compare the baseline characteristics between patients with NAFLD with developed ALF and those who did not develop ALF. Abbreviations: ALF, advanced liver fibrosis; AST, aspartate aminotransferase; ALT, alanine aminotransferase; CRP, C-reactive protein; CHO, carbohydrate; DBP, diastolic blood pressure; FPG, fasting plasma glucose; HDL, high-density lipoprotein; HOMA-IR, homeostatic model assessment for insulin resistance LDL, low-density lipoprotein; MET, metabolic equivalent of task; METS-IR, metabolic score for insulin resistance; NAFLD, non-alcoholic fatty liver disease; SBP, systolic blood pressure; TyG, triglyceride-glucose.

**Table 2:** Incidence of ALF during follow-up of participants with NAFLD.

Year Range	Follow-Up	Total (*n*)	Incidence Cases (*n*)	Incidence Rate Per 2 Years
2001–2002	Baseline	1368		
2003–2004	2 years	1368	33	2.41
2005–2006	4 years	1368	25	1.83
2007–2008	6 years	1368	31	2.27
2009–2010	8 years	1368	17	1.24
2011–2012	10 years	1368	34	2.49
2013–2014	12 years	1368	34	2.49
2015–2016	14 years	1368	43	3.14
2017–2018	16 years	1368	43	3.14

Abbreviations: ALF, advanced liver fibrosis; NAFLD, non-alcoholic fatty liver disease.

**Table 3:** Cox proportional hazard regression model for incident ALF according to the tertiles of each insulin resistance index.

	Total Cases, *n*	New Onset ALF Cases, *n*	Person-Years of Follow-Up	Incidence Rate Per 1000 Person-Years	Unadjusted		Model 1		Model 2		Model 3	
					HR (95% CI)	*p*	HR (95% CI)	*p*	HR (95% CI)	*p*	HR (95% CI)	*p*
	1368	260	19,939.4	13.0								
METS-IR												
T1 (<41.15)	456	99	6443.1	15.4	1 (reference)		1 (reference)		1 (reference)		1 (reference)	
T2 (41.15–45.70)	455	91	6670.2	13.6	0.89 (0.67–1.19)	0.429	0.82 (0.58–1.15)	0.250	0.81 (0.58–1.14)	0.235	0.82 (0.58–1.15)	0.250
T3 (≥45.71)	457	70	6826.1	10.3	0.67 (0.49–0.90)	0.009	0.63 (0.40–0.99)	0.047	0.60 (0.38–0.95)	0.030	0.59 (0.37–0.94)	0.026
TyG index												
T1 (<8.84)	456	98	6514.4	15.0	1 (reference)		1 (reference)		1 (reference)		1 (reference)	
T2 (8.84–9.26)	455	93	6645.0	14.0	0.93 (0.70–1.24)	0.620	0.88 (0.65–1.20)	0.410	0.92 (0.68–1.26)	0.616	0.96 (0.70–1.31)	0.774
T3 (≥9.27)	457	69	6780.0	10.2	0.68 (0.50–0.92)	0.014	0.66 (0.47–0.92)	0.013	0.73 (0.52–1.03)	0.071	0.74 (0.53–1.04)	0.087
HOMA-IR												
T1 (<1.91)	457	84	6685.6	12.6	1 (reference)		1 (reference)		1 (reference)		1 (reference)	
T2 (1.91–2.65)	456	82	6648.3	12.3	0.98 (0.73–1.33)	0.919	1.08 (0.78–1.48)	0.654	1.07 (0.77–1.47)	0.697	1.16 (0.81–1.54)	0.505
T3 (≥2.65)	455	94	6605.5	14.2	1.15 (0.86–1.55)	0.347	1.04 (0.75–1.44)	0.811	1.02 (0.74–1.40)	0.920	1.04 (0.75–1.44)	0.826

Model 1: adjusted for sex, age, body mass index, physical activity, smoking status, drinking status, total energy intake, and vitamin E intake. Model 2: adjusted for variables used in Model 1 plus mean blood pressure, serum total cholesterol level, and serum CRP level. Model 3: adjusted for variables used in Model 2 plus serum ALT level. Abbreviations: ALF, advanced liver fibrosis; HR, hazard ratio; CI, confidence interval; METS-IR, metabolic score for insulin resistance; TyG, triglyceride-glucose; HOMA-IR, homeostatic model assessment for insulin resistance; CRP, C-reactive protein; ALT, alanine aminotransferase.

## Data Availability

The dataset used in this study (the KNHANES and Ansan-Ansung cohort) can be provided after review and evaluation of the research plan by the Korea Centers for Disease Control and Prevention (http://www.cdc.go.kr/CDC/eng/main.jsp) (accessed on 14 January 2022).

## Supplementary Materials

The following supporting information can be downloaded at: https://www.mdpi.com/article/10.3390/nu14153039/s1, Table S1: Baseline characteristics of participants with NAFLD without ALF at baseline who were followed-up at least once after the baseline survey. Abbreviation: NAFLD, non-alcoholic fatty liver disease; ALF, advanced liver fibrosis; Table S2: Cox proportional hazards regression model for incident ALF of three different insulin resistant indices. Abbreviation: ALF, advanced liver fibrosis; Table S3: Cox proportional hazards regression model for incident ALF of three different insulin resistant indices according to the presence of diabetes. Figure S1: Flow chart of the study population. Abbreviation: NAFLD, non-alcoholic fatty liver disease; ALF, advanced liver fibrosis; KoGES, Korean Genome and Epidemiology Study; METS-IR, metabolic score for insulin resistance, TyG, triglyceride-glucose; HOMA-IR, homeostatic model assessment for insulin resistance; Figure S2: Longitudinal changes in serum triglyceride level according to the METS-IR tertiles. Abbreviation: METS-IR, metabolic score for insulin resistance; Figure S3: Longitudinal changes in body mass index according to advanced liver fibrosis status; Figure S4: Longitudinal changes in body mass index according to the METS-IR tertiles. Abbreviations: METS-IR, metabolic score for insulin resistance.
