# Supplementary material for: Metabolic Score for Insulin Resistance Is Inversely Related to Incident Advanced Liver Fibrosis in Patients with Non-Alcoholic Fatty Liver Disease
Source: Nutrients. 2022 Jul 24;14(15):3039. doi: 10.3390/nu14153039 (PMC9330359; doi:10.3390/nu14153039)
Supplement: Supplementary file 1 [file nutrients-14-03039-s001.zip › nutrients-1799357-supplementary.pdf]

nutrients

Table S1. Baseline characteristics of participants with NAFLD with or without ALF at the baseline survey.

Variables	Without ALF	With ALF	Total	*p**
Number of participants, n	2148	70	2218	
Male sex, n (%)	911 (42.4%)	47 (67.1%)	958 (43.2%)	< 0.001
Age, years	54.1 ± 8.7	60.1 ± 7.2	54.3 ± 8.7	< 0.001
Waist circumference	89.0 ± 7.6	84.3 ± 9.8	88.9 ± 7.7	< 0.001
Body mass index, kg/m^2	26.6 ± 3.0	23.5 ± 3.7	26.5 ± 3.0	< 0.001
MBP, mmHg	102.8 ± 12.4	102.4 ± 13.5	102.8 ± 12.4	0.811
Current drinker, n (%)	793 (37.3%)	40 (59.7%)	833 (38.0%)	< 0.001
Smoking status, n (%)				< 0.001
Never smoker	1350 (63.8%)	27 (40.3%)	1377 (63.0%)	
Former smoker	318 (15.0%)	16 (23.9%)	334 (15.3%)	
Someday smoker	47 (2.2%)	5 (7.5%)	52 (2.4%)	
Everyday smoker	402 (19.0%)	19 (28.4%)	421 (19.3%)	
Physical activity				0.482
< 7.5 METs-hr/week	205 (10.0%)	5 (7.7%)	210 (9.9%)	
7.5–30 METs-hr/week	1258 (61.2%)	37 (56.9%)	1295 (61.1%)	
≥ 30 METs-hr/week	592 (28.8%)	23 (35.4%)	615 (29.0%)	
Platelets, /mm^3	279.2 ± 63.5	142.9 ± 66.0	274.9 ± 67.9	< 0.001
FPG, mg/dL	96.4 ± 29.9	100.1 ± 33.3	96.5 ± 30.0	0.303
Insulin, μIU/mL	10.1 [7.6;12.7]	7.3 [5.2;11.6]	10.1 [7.5;12.6]	< 0.001
Total cholesterol, mg/dL	201.6 ± 35.9	170.5 ± 41.0	200.6 ± 36.5	< 0.001
triglyceride, mg/dL	189.0 [146.0;258.0]	139.5 [104.0;196.0]	187.0 [144.0;256.0]	< 0.001
HDL cholesterol, mg/dL	40.1 ± 8.2	46.5 ± 13.9	40.3 ± 8.5	< 0.001
LDL cholesterol, mg/dL	120.7 ± 32.8	89.7 ± 36.5	119.7 ± 33.3	< 0.001
AST, U/L	28.0 [24.0;35.5]	75.5 [47.0;117.0]	29.0 [24.0;36.0]	< 0.001
ALT, U/L	30.0 [22.0;44.0]	49.0 [36.0;77.0]	31.0 [23.0;45.0]	0.007
Gamma-GTP, U/L	27.0 [16.0;48.0]	116.0 [32.0;329.0]	27.0 [16.0;49.0]	<0.001
Total bilirubin, mg/dL	0.5 [0.4; 0.7]	0.6 [0.4; 0.9]	0.5 [0.4; 0.7]	0.001
Albumin, g/L	4.2 ± 0.3	4.0 ± 0.4	4.2 ± 0.3	<0.001
CRP, mg/dL	0.19 [0.10;0.31]	0.13 [0.05;0.31]	0.18 [0.10;0.31]	0.085
Total caloric intake, kcal/day	1978.1 ± 729.3	1943.7 ± 810.7	1977.1 ± 731.8	0.705
CHO intake, g/day	353.6 ± 127.6	347.4 ± 143.1	353.4 ± 128.1	0.697
Protein intake, g/day	66.2 ± 29.4	63.9 ± 31.0	66.2 ± 29.4	0.525
Fat intake, g/day	31.1 ± 20.6	30.5 ± 21.6	31.1 ± 20.6	0.821
Vitamin E intake, mg/day	9.6 ± 5.8	8.5 ± 5.1	9.6 ± 5.8	0.140
Diabetes mellitus, n (%)	671 (31.2%)	28 (40.0%)	699 (31.5%)	0.155
Hypertension, n (%)	1329 (61.9%)	42 (60.0%)	1371 (61.8%)	0.848
Dyslipidemia, n (%)	1579 (73.5%)	34 (48.6%)	1613 (72.7%)	< 0.001
Fibrosis-4 score	1.0 [0.8; 1.3]	3.9 [3.1; 6.7]	1.0 [0.8; 1.4]	< 0.001
METS-IR	43.52 ± 5.98	36.82 ± 8.41	43.31 ± 6.18	< 0.001
TyG index	9.11 ± 0.53	8.89 ± 0.69	9.11 ± 0.54	0.008
HOMA-IR	2.64 ± 2.05	2.14 ± 1.60	2.62 ± 2.04	0.013

* *p*-value for comparison of the baseline characteristics between patients with NAFLD with and without ALF. **Abbreviations:** NAFLD, non-alcoholic fatty liver disease; ALF, advanced liver fibrosis; SBP, systolic blood pressure; DBP, diastolic blood pressure; MET, metabolic equivalent of task; FPG, fasting plasma glucose; HDL, high-density lipoprotein; LDL, low-density lipoprotein; AST, aspartate aminotransferase; ALT, alanine aminotransferase; CRP, C-reactive protein; CHO, carbohydrate; METS-IR, metabolic score for insulin resistance; TyG, triglyceride-glucose; HOMA-IR, homeostatic model assessment for insulin resistance.

Table S2. Cox proportional hazards regression model for incident ALF of three different insulin resistance indices.

	Numbers, n	New-onset ALF, n	Person-years of follow-up	Incidence rate per 1000 person-years	Unadjusted		Model 1		Model 2		Model 3	
					HR (95% CI)	*p*	HR (95% CI)	*p*	HR (95% CI)	*p*	HR (95% CI)	*p*
	1368	260	19939.4	13.0								
METS-IR (per 1 increment)					0.97 (0.95–0.99)	0.003	0.93 (0.90–0.97)	0.001	0.92 (0.88–0.96)	< 0.001	0.92 (0.88–0.96)	0.001
TyG index (per 1 increment)					0.71 (0.56–0.91)	0.007	0.64 (0.49–0.84)	0.001	0.64 (0.47–0.87)	0.004	0.66 (0.49–0.89)	0.007
HOMA-IR (per 1 increment)					1.02 (0.98–1.06)	0.844	1.02 (0.95–1.10)	0.582	1.02 (0.94–1.10)	0.700	1.03 (0.95–1.11)	0.504

Model 1: adjusted for sex, age, body mass index, physical activity, smoking status, drinking status, total caloric intake, and vitamin E intake. Model 2: adjusted for variables used in Model 1 plus mean blood pressure, serum total cholesterol level, and serum CRP level. Model 3: adjusted for variables used in Model 2 plus serum ALT level. Abbreviations: ALF, advanced liver fibrosis; HR, hazard ratio; CI, confidence interval; METS-IR, metabolic score for insulin resistance; TyG, triglyceride-glucose; HOMA-IR, homeostatic model assessment for insulin resistance; CRP, C-reactive protein; ALT, alanine aminotransferase.

Table S3. Cox proportional hazards regression model for incident ALF of three different insulin resistant indices according to the presence of diabetes.

	Unadjusted model		Fully adjusted model	
	HR (95% CI)*	*p*	HR (95% CI)*	*p*
**DM subgroup**				
METS-IR				
T1 (< 41.15)	1 (reference)		1 (reference)	
T2 (41.15–45.70)	0.80 (0.47–1.36)	0.415	0.86 (0.44–1.66)	0.645
T3 (≥ 45.71)	0.59 (0.35–1.01)	0.056	0.55 (0.23–1.33)	0.183
TyG index				
T1 (< 8.84)	1 (reference)		1 (reference)	
T2 (8.84–9.26)	1.38 (0.75–2.53)	0.301	1.14 (0.57–2.28)	0.710
T3 (≥ 9.27)	0.86 (0.47–1.56)	0.623	0.92 (0.47–1.79)	0.796
HOMA-IR				
T1 (< 1.91)	1 (reference)		1 (reference)	
T2 (1.91–2.65)	1.21 (0.67–2.18)	0.537	1.34 (0.70–2.56)	0.376
T3 (≥ 2.65)	0.97 (0.57–1.68)	0.925	0.93 (0.50–1.70)	0.803
**Non-DM subgroup**				
METS-IR				
T1 (< 41.15)	1 (reference)		1 (reference)	
T2 (41.15–45.70)	0.94 (0.67–1.32)	0.415	0.75 (0.50–1.15)	0.186
T3 (≥ 45.71)	0.70 (0.48–1.01)	0.059	0.56 (0.32–0.99)	0.046
TyG index				
T1 (< 8.84)	1 (reference)		1 (reference)	
T2 (8.84–9.26)	0.81 (0.59–1.13)	0.215	0.84 (0.58–1.21)	0.839
T3 (≥ 9.27)	0.59 (0.40–0.88)	0.009	0.61 (0.39–0.94)	0.025
HOMA-IR				
T1 (< 1.91)	1 (reference)		1 (reference)	
T2 (1.91–2.65)	0.92 (0.65–1.32)	0.659	1.03 (0.70–1.50)	0.897

T3 (≥ 2.65)	1.24 (0.87–1.77)	0.232	1.02 (0.69–1.53)	0.908

*Variables including sex, age, body mass index, physical activity, smoking status, drinking status, total calorie intake, vitamin E intake, mean blood pressure, serum total cholesterol level, serum CRP level, and serum ALT level were used in the fully adjusted model. Abbreviations: DM, diabetes mellitus; HR, hazard ratio; CI, confidence interval; METS-IR, metabolic score for insulin resistance; TyG, triglyceride-glucose; HOMA-IR, homeostatic model assessment for insulin resistance; CRP, C-reactive protein; ALT, alanine aminotransferase.

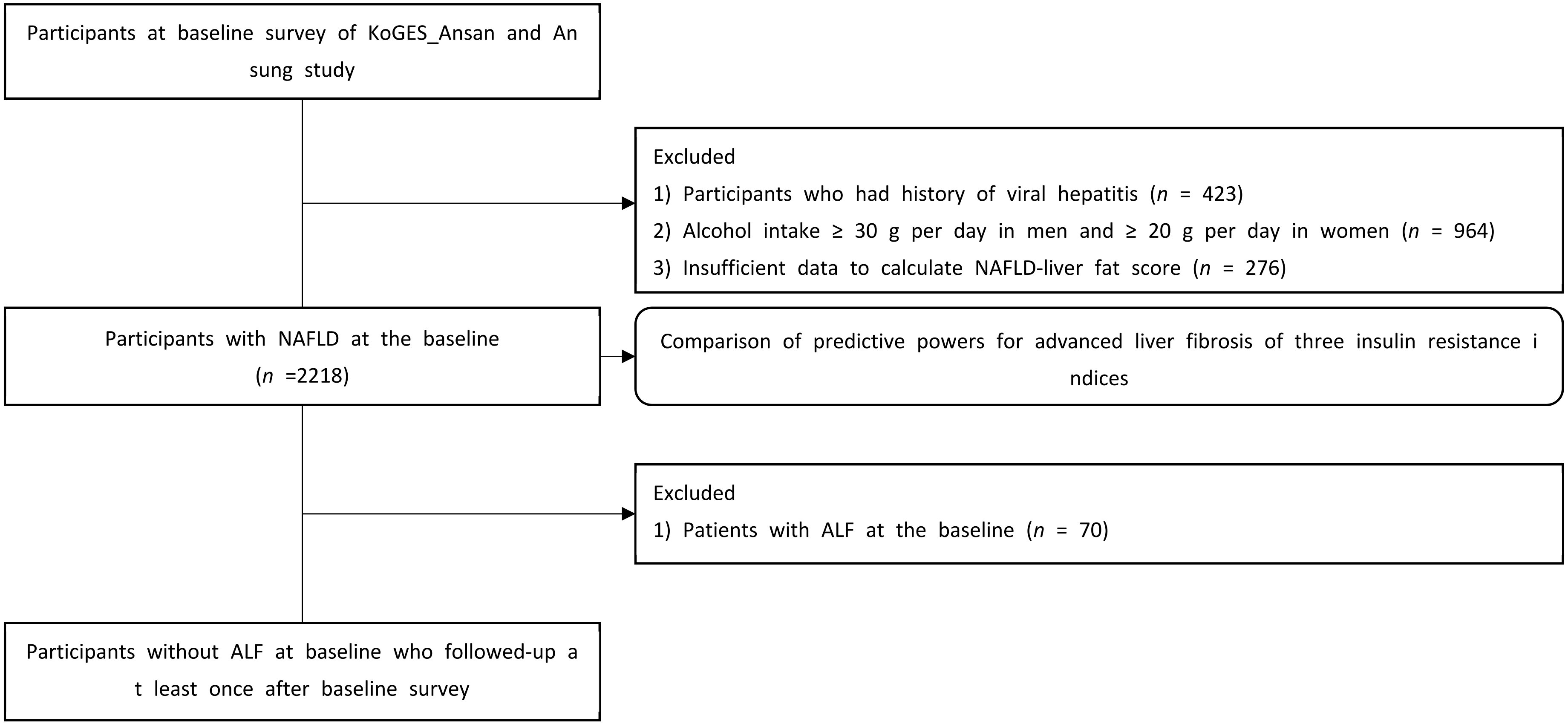
Participants at baseline survey of KoGES_Ansan and An sung study
Excluded
1) Participants who had history of viral hepatitis (n = 423)
2) Alcohol intake ≥ 30 g per day in men and ≥ 20 g per day in women (n = 964)
3) Insufficient data to calculate NAFLD-liver fat score (n = 276)
Participants with NAFLD at the baseline (n =2218)
Comparison of predictive powers for advanced liver fibrosis of three insulin resistance i ndices
Excluded
1) Patients with ALF at the baseline (n = 70)
Participants without ALF at baseline who followed-up a t least once after baseline survey

Figure S1. Flow chart of the study population. Abbreviation: NAFLD, non-alcoholic fatty liver disease; ALF, advanced liver fibrosis; KoGES, Korean Genome and Epidemiology Study; METS-IR, metabolic score for insulin resistance, TyG, triglyceride-glucose; HOMA-IR, homeostatic model assessment for insulin resistance

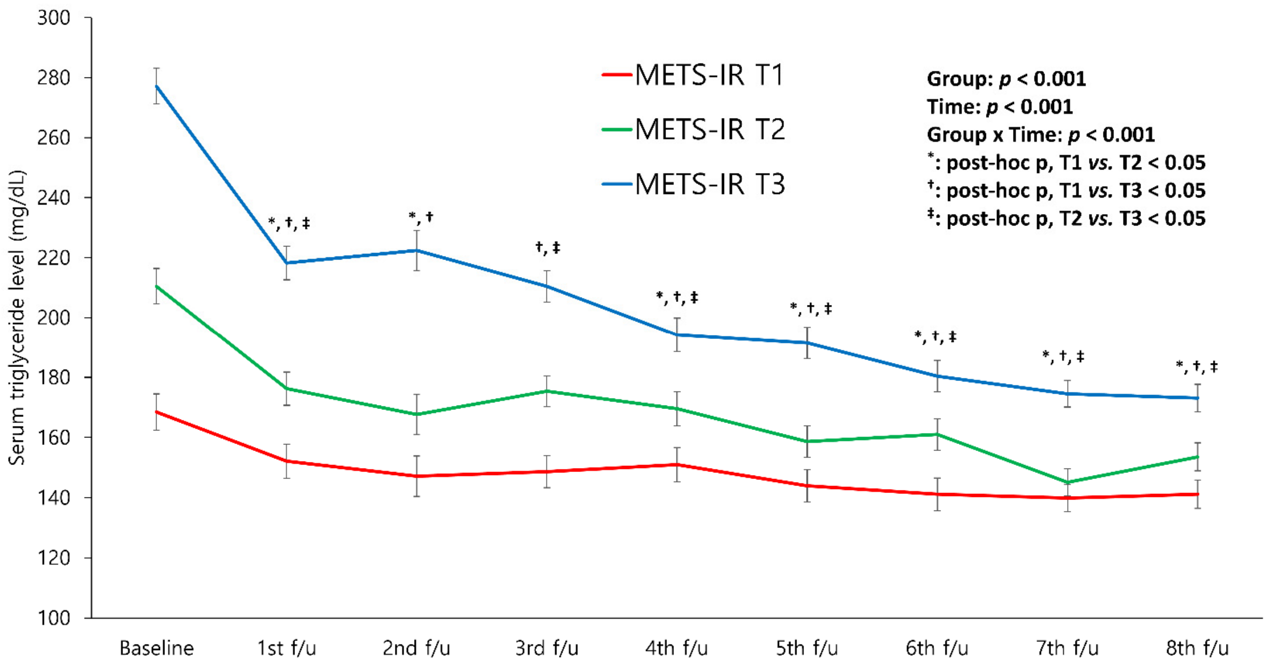

Figure S2. Longitudinal changes in serum triglyceride level according to the METS-IR tertiles. Abbreviation: METS-IR, metabolic score for insulin resistance.

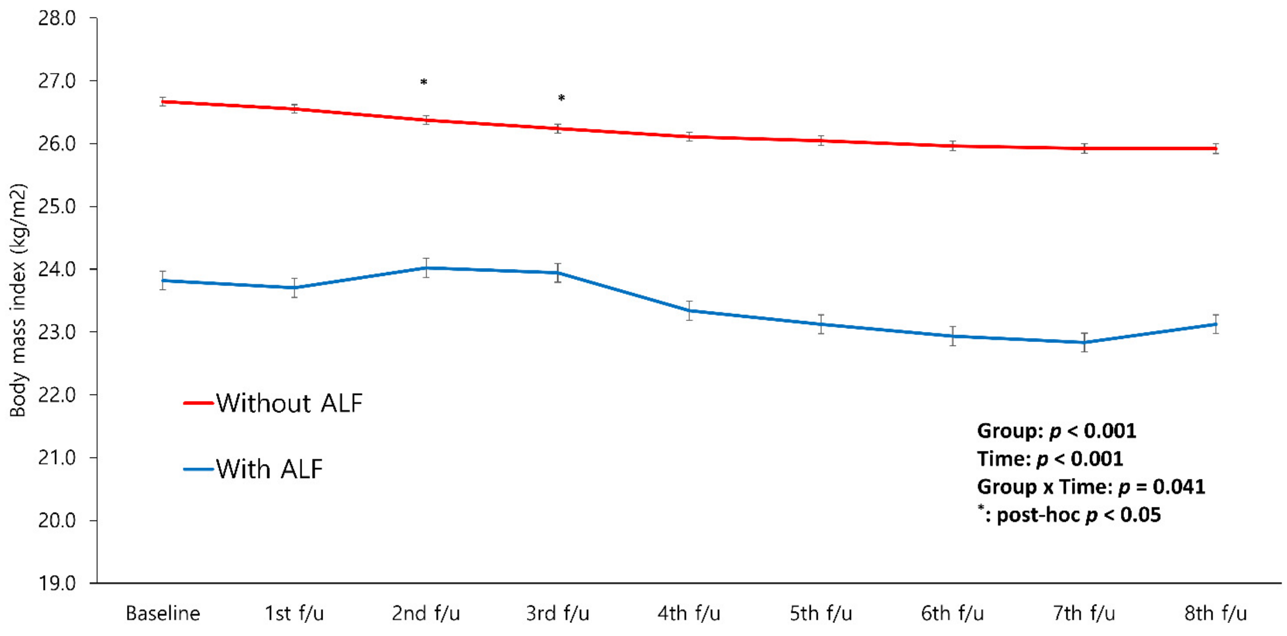

Figure S3. Longitudinal changes in body mass index according to advanced liver fibrosis status.

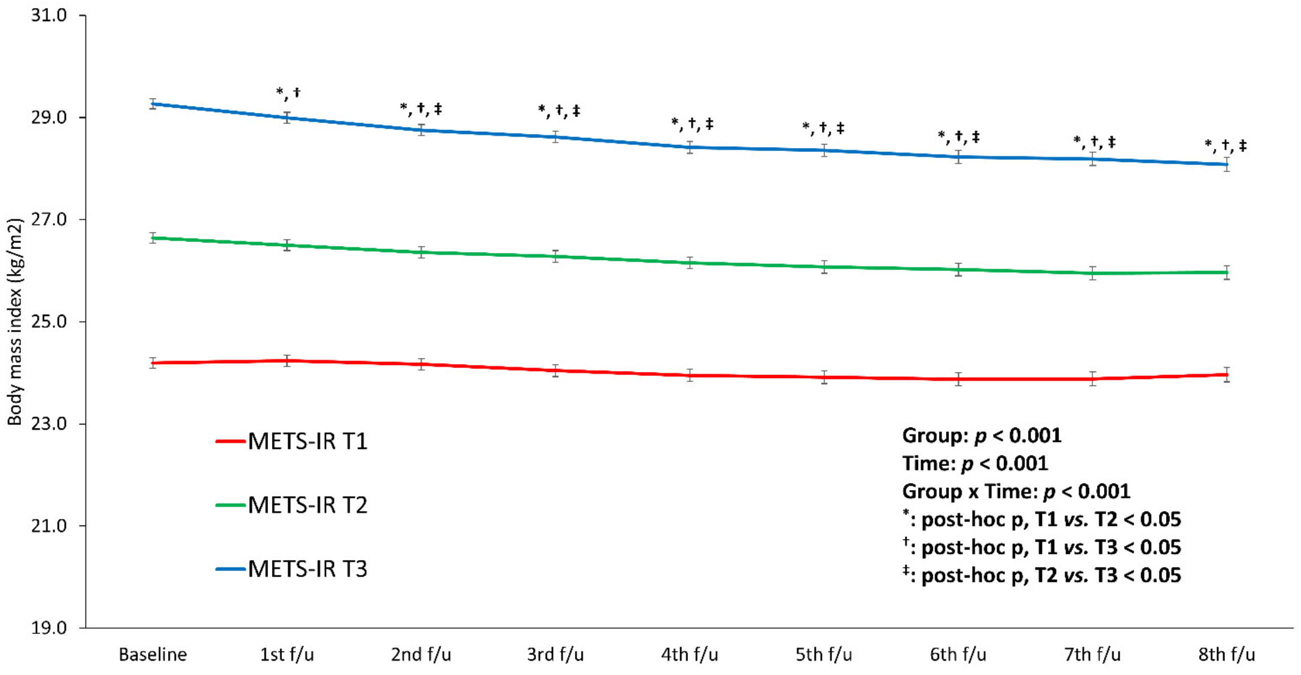

Figure S4. Longitudinal changes in body mass index according to the METS-IR tertiles. Abbreviations: METS-IR, metabolic score for insulin resistance.
